# An Indoor Monitoring System for Ambient Assisted Living Based on Internet of Things Architecture

**DOI:** 10.3390/ijerph13111152

**Published:** 2016-11-17

**Authors:** Gonçalo Marques, Rui Pitarma

**Affiliations:** Unit for Inland Development, Polytechnic Institute of Guarda, Avendia Doutor Francisco Sá Carneiro N° 50, 6300-559 Guarda, Portugal; rpitarma@ipg.pt

**Keywords:** indoor air quality, indoor environment, air quality monitoring, wireless sensor network, ZigBee, gas sensors, smart cities, ambient assisted living, Internet of Things

## Abstract

The study of systems and architectures for ambient assisted living (AAL) is undoubtedly a topic of great relevance given the aging of the world population. The AAL technologies are designed to meet the needs of the aging population in order to maintain their independence as long as possible. As people typically spend more than 90% of their time in indoor environments, indoor air quality (iAQ) is perceived as an imperative variable to be controlled for the inhabitants’ wellbeing and comfort. Advances in networking, sensors, and embedded devices have made it possible to monitor and provide assistance to people in their homes. The continuous technological advancements make it possible to build smart objects with great capabilities for sensing and connecting several possible advancements in ambient assisted living systems architectures. Indoor environments are characterized by several pollutant sources. Most of the monitoring frameworks instantly accessible are exceptionally costly and only permit the gathering of arbitrary examples. iAQ is an indoor air quality system based on an Internet of Things paradigm that incorporates in its construction Arduino, ESP8266, and XBee technologies for processing and data transmission and micro sensors for data acquisition. It also allows access to data collected through web access and through a mobile application in real time, and this data can be accessed by doctors in order to support medical diagnostics. Five smaller scale sensors of natural parameters (air temperature, moistness, carbon monoxide, carbon dioxide, and glow) were utilized. Different sensors can be included to check for particular contamination. The results reveal that the system can give a viable indoor air quality appraisal in order to anticipate technical interventions for improving indoor air quality. Indeed indoor air quality might be distinctively contrasted with what is normal for a quality living environment.

## 1. Introduction

Ambient assisted living (AAL) is an emerging multi-disciplinary field aiming at providing an ecosystem of different types of sensors, computers, mobile devices, wireless networks, and software applications for personal healthcare monitoring and telehealth systems [1]. Indoor environments are characterized by several pollutant sources. Accordingly, indoor air quality (iAQ) is perceived as a vital variable to be controlled for the inhabitants’ wellbeing and solace. This issue is more important if we take into consideration that today most people spend more than 90% of their time in artificial environments [2], and health problems and diseases caused by poor indoor air quality can negatively affect worker performance and productivity. According to the United States Environmental Protection Agency [3], human exposure to indoor air contaminations might be 2 to 5 times—once in a while more than 100 times—higher than outdoor toxin levels, because a home’s interior accumulates and concentrates pollutants given off by finishes, furnishings, and the daily activities of the occupants [4]. Indeed, indoor air toxins have been positioned among the main five ecological dangers to general wellbeing. Ventilation is used in buildings to create thermally comfortable environments with acceptable iAQ by regulating indoor air parameters, such as air temperature, relative humidity, air speed, and chemical species concentrations in the air [5]. In this study, the authors present some numerical predictions of pollutant dispersion in a ventilated room.

An indoor air quality assessment system helps in the detection and improvement of indoor air quality. Local and distributed assessment of chemical concentrations is significant for safety (e.g., gas spills detection and pollution monitoring) and security applications as well as to control heating, ventilation, and air conditioning (HVAC) systems for energy efficiency [6]. In fact, the indoor air quality measured in the manufactured environment gives a consistent stream of data for reliable management of building automation systems and provides a platform for informed decision-making [7]. However, the monitoring systems presently available are normally very expensive and only allow for the collection of random samples.

Recently, several new systems have been developed for monitoring environmental parameters, always with the aim of improving indoor air quality efficiency [8]. Actually, the availability of affordable sensors, processing units, radios, and Wi-Fi communication technologies integrated in a single board is leading to the use of wireless communications and computing for interacting with the physical world in applications such as air quality control [9]. Wireless indoor air quality monitoring in order to provide real-time information for assisted living is proposed in [7], a solution using various gas sensors in order to monitor methane, propane, and carbon dioxide and monoxide. Another study involving wireless sensor networks for indoor air quality monitoring was proposed in [10]. Challenges in designing and implementing of an effective ambient assisted living systems include information architecture, interaction design, human-computer interaction, ergonomics, usability, and accessibility [11]. There are also social and ethical problems, such as the acceptance by older adults, and the privacy and confidentiality that should be a requisite of all AAL devices. In fact, it is also important to ensure that technology does not replace human care but presents solutions that complement and support healthcare treatments and systems. In 2050, 20% of the world population will be aged 60 or older [12], which will result in an increase of diseases, health care costs, shortage of care givers, dependency, and a harsher social impact. It is a fact that 87% of people prefer to stay in their homes and support the huge cost of nursing care [13].

This study describes the iAQ system, developed by the authors, which aims to ensure, autonomously, accurately, and simultaneously, the indoor air quality monitoring of different building rooms. iAQ is an indoor air quality system based on an IoT (Internet of Things) paradigm that incorporates in its construction Arduino, ESP8266, and XBee technologies for processing and data transmission and micro sensors for data acquisition. It also allows access to data collected from different places simultaneously through web access and through mobile applications in real time.

## 2. Materials and Methods

Smart homes have been researched for decades, the first project of Smart Rooms was implemented by the MIT Media Lab (Cambridge, MA, USA) [14], and today three major categories of smart homes exist. The first category detects and recognizes the actions of its residents to determine their health. The second category aims at the storage and retrieval of multimedia captured within the smart home, in different levels from photos to experiences. The third category is surveillance, where the data captured in the environment are processed to obtain information that can help to raise alarms in order to protect the home and the residents. There is also a type of smart home that has the objective of reducing energy consumption by monitoring and controlling electric devices [15].

Recent advances in information technology has allowed for lower prices of smart homes, but providing them with intelligence environments to make complex decisions remains a challenge. In the future, smart home numbers will increase with the use of sensors that will put the data acquired in monitoring databases in real time.

Three broad views are introduced in [16]: A functional view, an instrumental view, and a socio-technical view. The functional view sees smart homes as a way of better managing the demands of daily living through technology. The instrumental vision reinforces the potential of smart houses in contributing to the improvement of household energy efficiency, as well as their contribution towards reducing carbon dioxide emissions. In addition, smart houses help with the healthcare requirements of patients. The socio-specialized perspective sees the shrewd home as the following influx of advancement in the continuous jolt and digitalization of regular life.

One interesting smart home application is introduced in [17] called Vital-Radio, a wireless sensing technology that monitors breathing and heart rate without body contact. In Europe, smart home projects include iDorm [18], Gloucester Smart Home [19], and CareLab [20].

The iAQ system is also compatible and can be fully integrated into smart houses, but also in normal houses, as it has been developed based on Internet of Things architecture, which is a suitable approach to building support solutions for healthcare.

### 2.1. Implementation

The iAQ system is an automatic indoor air quality monitoring system that allows the user, such as the building manager, to know, in real time, a variety of environmental parameters such as air temperature, relative humidity, carbon monoxide (CO), carbon dioxide (CO_2_), and luminosity. Other sensors for specific pollutants can be added.

The parameters are monitored using the iAQ Sensor system, which collects data and sends it to the iAQ Gateway system that records the data in a MySQL (Oracle, Redwood, CA, USA) database using web services developed in Hypertext Preprocessor (PHP).

The end user can access the data from the web portal iAQ Web built in PHP. After login, the end user can access the iAQ Web and can get all the information about environmental parameters. The monitoring data are shown as numeric values or in a chart form. This portal also allows the user to keep the parameters history. Providing a history of changes, the system helps the user to analyze precisely and detail air quality behavior. This is very important for deciding on possible interventions to improve the air quality in the building. The iAQ Web is also equipped with a powerful alert manager that advises the user when a specific parameter exceeds the maximum value. In order to allow quick, simple, and intuitive access anytime and anywhere, an application for Android smartphones named iAQMobile, described in Section 2.3, has also been created.

### 2.2. Wireless Sensor Network Architecture

The wireless communication is implemented using the XBee module, which implements the IEEE 802.15.4 radio and ZigBee networking protocol [21]. The IEEE 802.15.4 standard specifies the physical and medium access control layers for low data-rate wireless personal area networks. ZigBee is a low-cost, low-power, wireless mesh networking standard built upon 802.15.4 [22,23].

Communication signals are transmitted from the iAQ Sensor to the base station iAQ Gateway use XBee. The modules operate within the 2.4 GHz frequency band and outdoor RF (Radio Frequency) line-of-sight range up to 4000 ft (1200 m) and RF data rate 250,000 bps. These modules use the IEEE 802.15.4 networking protocol for fast point-to-multipoint or peer-to-peer networking. They are designed for high-throughput applications requiring low latency and predictable communication timing. XBee modules are ideal for low-power, low-cost applications. XBee-PRO modules are power-amplified versions of XBee modules for extended-range applications [24].

**Figure 1:**
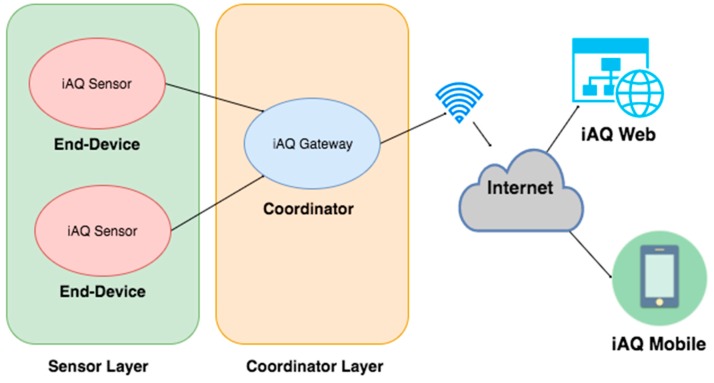
Wireless sensor networks (WSN) architecture.

The wireless sensor networks (WSN) have has a star topology as its network configuration and uses single-hop communication due to the fact that the endpoints are relatively close and communicate with a single coordinator node (Figure 1).

The current consumption of the WSN sensor and coordinator nodes are measured in order to analyze the lifetime of the WSN, and the results are presented in Table 1. In order to promote energy efficiency, the system is placed into sleep mode whenever possible. Considering 10% of iAQ Sensor time in transmission state, 5% in receiving state, and 85% in sleeping state, the medium consumption values are 126 mA. Considering 5% of iAQ Gateway time is in transmitting transmission state, 10% in receiving state and 85% in sleeping state, the medium consumption values are 72 mA. Thus, using 10,000 mAh, the iAQ Sensor’s battery lifetime is estimated to be 79 h, and the iAQ Gateway’s is estimated at 139 h.

**Figure 2:**
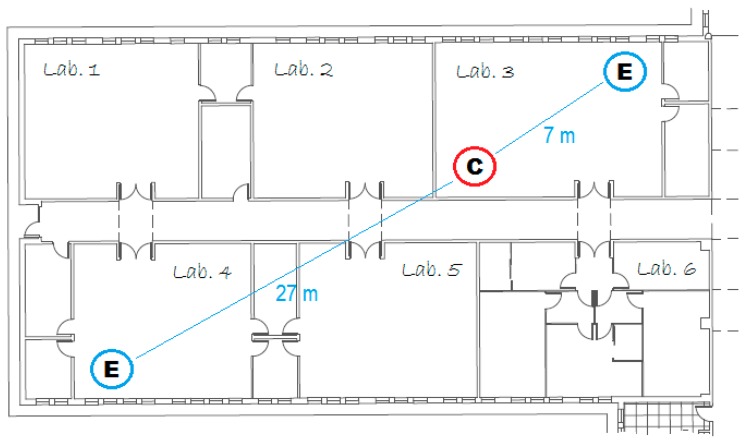
Polytechnic Institute of Guarda Laboratory plan.

The maximum transmission range between iAQ Sensor and iAQ Gateway was measured at the Polytechnic Institute of Guarda Laboratories Floor using two end-devices and one coordinator as represented in Figure 2. The maximum distance between the end-device and the coordinator is about 27 m considering obstacles between the nodes. In Figure 2, the iAQ Gateaway (Coordinator) is represented by the letter C, and the iAQ Sensor End-device is represented by the letter E.

**Figure 3:**
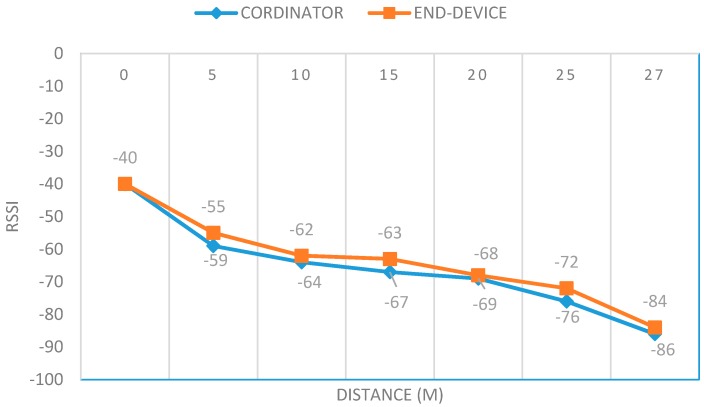
Relation between distance and RSSI.

The measured RSSI values (Received Signal Strength Indication) of the communication between the nodes is shown in Figure 3 as a function of the distance. The RSSI values are represented in dBm, and the orange line represents the RSSI values measured in the coordinator (iAQ Gateway) and the end-device (iAQ Sensor).

### 2.3. iAQ Mobile

Presently, smartphones have incredible processing capabilities as well as a set of very interesting sensors for the study of assisted living such as GPS (Global Position System), accelerometer, gyroscope, proximity sensor, camera, microphone, NFC (Near Field Communication), and BLE (Bluetooth Low Energy).

Smartphones now have mobile sensors that perform activity recognition and detect physical activities such as walking, running, climbing stairs, descending stairs, driving, cycling, and being inactive with no additional sensing hardware [25,26].

There are also projects that can detect driver behaviour as safe or unsafe using optimal path detection algorithms and Bayesian classification where event data are acquired by smart phone sensors via accelerometer, gyroscope, and magnetometer [27].

Today, the smartphone has a key role in building smart communications architectures for ambient assisted living to know what is happening in the network and to detect if elderly people need assistance, as proposed in [28].

With regard to the importance of the smartphone’s role in human life, an Android application was created. This application provides data authentication and protection mechanisms for information visualizations and allows one to view system data in detail and receive notifications when any of the values exceed regular values. The mobile application is designed to provide quick and easy access to the iAQ system to allow the end user to keep all the relevant information of the iAQ system in your pocket.

This application was built for the Android system using the IDE (Integrated Development Environment) AndroidStudio and has the minimum requirements API (Application Programming Interface) 15: Android 4.0.3 Ice Cream Sandwich in order to be compatible with 96.2% of active devices in the Google Play Store (information collected on 22 January 2016).

**Figure 4:**
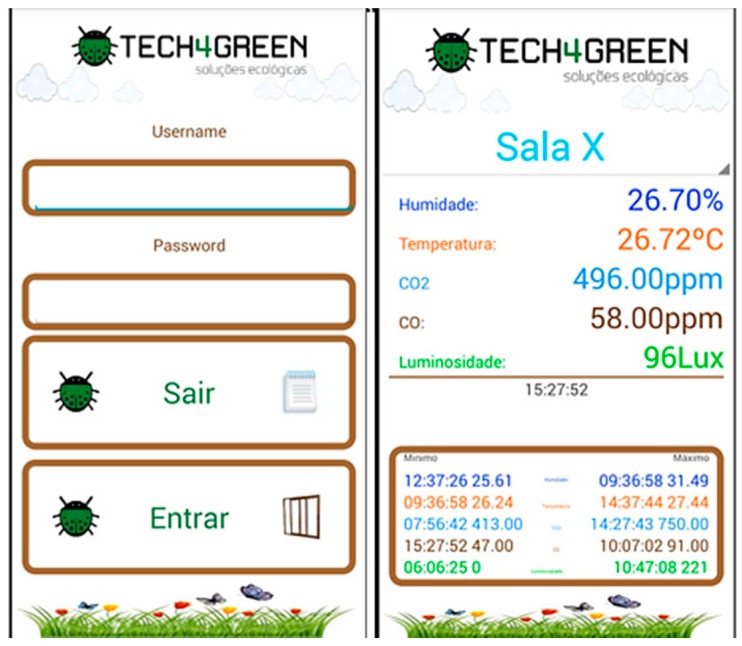
Android app.

In Figure 4, two screens of the mobile application are shown: the left shows the login screen in the application that ensures access only to authorized users. On the right image, the login form where the user can select one of their iAQ Sensor nodes is shown and the current humidity, temperature, carbon dioxide, carbon monoxide, and light values will be automatically displayed, and the user can also quickly access the minimum and maximum values of these parameters and the dates of these incidents.

### 2.4. Hardware and System Architecture

**Figure 5:**
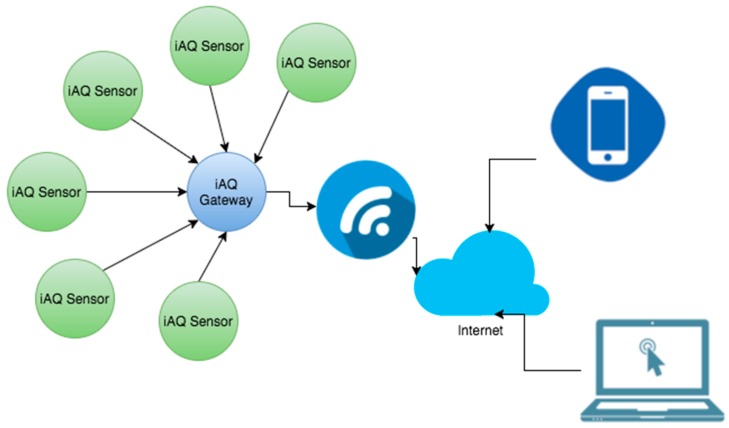
iAQ system architecture.

The iAQ system is composed of one or several iAQ Sensors. They are used to collect and transfer environmental factors from the different rooms where they are installed. The iAQ Sensors send the data to the iAQ Gateway (Figure 5), which is connected to the Internet via Wi-FI 2.4 Ghz in order to record data in real-time to a structured database.

Therefore, it is possible to construct a modular system that can monitor one or more spaces simultaneously. Figure 5 schematically illustrates the system architecture used in the iAQ.

The iAQ Sensor is built using the embedded Arduino Mega system, an open source platform that incorporates an Atmel AVR microcontroller (Atmel, San José, CA, USA) [29,30]. In order to allow communication between the iAQ Sensor and iAQ Gateway, ZigBee technology was applied with the use of Xbee modules.

A truly interesting example of IoT combined with AAL is proposed in [31], where an integrated platform for the monitoring and controlling of households that uses a ZigBee Wireless network is presented.

**Figure 6:**
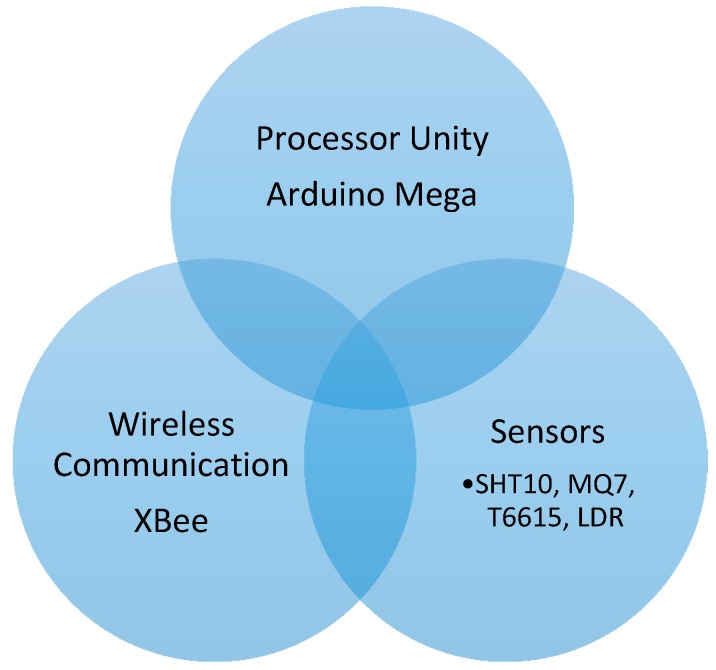
iAQ Sensor.

**Figure 7:**
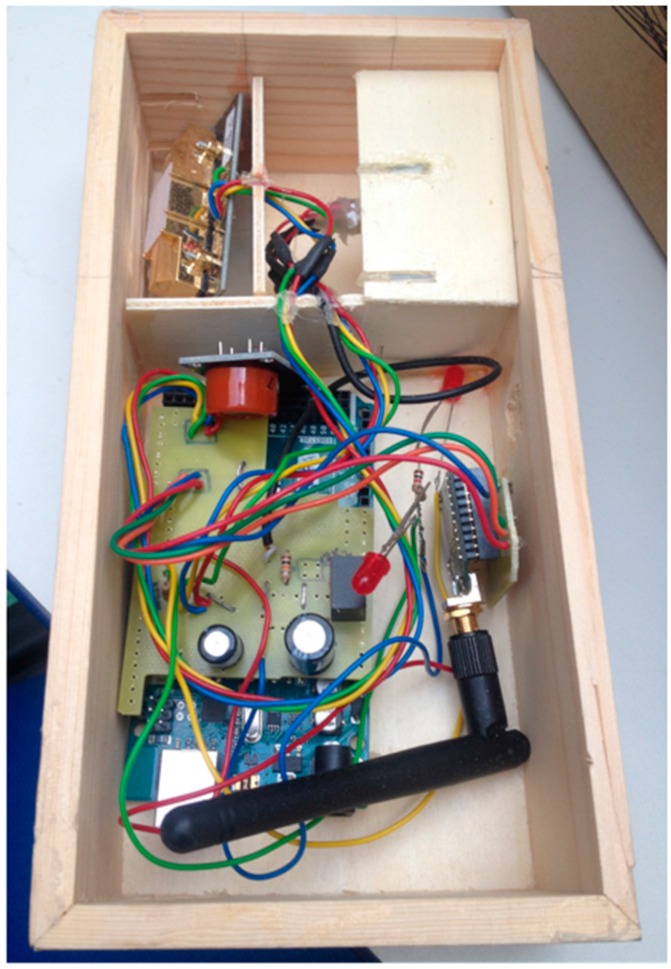
iAQ Sensor hardware.

The iAQ Sensor is equipped with multiple sensors, a processing unit (Arduino MEGA, a wireless communication, and a mesh networking module as schematically shown in Figure 6 (see also [32]). Currently, the iAQ Sensor is equipped with five sensors (Figure 7): air temperature, relative humidity (RH), carbon monoxide (CO), carbon dioxide (CO_2_), and luminosity.

A brief description of the sensors used is presented below:

- Sensor SHT10—a low power, stable, and fully calibrated relative humidity and temperature sensor [33]. Measurement range: 0%–100% (humidity), −40–120 °C (temperature). Accuracy: ±4.5% (humidity), ±0.5 °C (temperature). Response time <30 s.
- MQ7 Sensor—a high sensitivity CO (carbon monoxide) sensor with several features [34]: high sensitivity, fast response, a wide detection range (20 to 2000 ppm), stable performance and long life, simple drive circuit; requires manual calibration.
- T6615 CO_2_ Sensor—a low power, good performance CO_2_ (carbon dioxide) sensor (designed for HVAC purposes) with the following main specifications [35]—Measurement range: 0–5000 ppm. Accuracy: ±50 ppm ±3% of Reading. Response time: 2 min. Automatic calibration (every 24 h).
- LDR 5 mm Sensor—a sensor that allows the detection of light; it is basically a resistor that changes its resistive value (in ohms) depending on how much light is shining onto the squiggly face [36]. Since it is low cost but inaccurate, it should not be used to try to determine precise light levels in lux; instead, we can expect only to be able to determine basic light changes. Resistance range: 200 Kohm (dark) to 10 Kohm (10 lux brightness). Sensitivity range: CdS cells respond to light between 400 nm (violet) and 600 nm (orange) wavelengths, peaking at about 520 nm (green).

The iAQ Gateway uses Wemos Mini D1 (Wemos Electronics) as a processing unit. This microcontroller is a mini Wi-Fi board based on ESP-8266EX that provides 11/1 digital input/output pins, 1 analogue input, and a micro USB connection for programming and power. Wemos Mini D1 is fully compatible with Arduino IDE, has an 80/160 MHZ clock speed, 4 MB Flash, a 3.3 V operating voltage, a reduced size of 34.2 mm × 25.6 mm, and a weight of 10 g. Weemos Mini D1 have integrated Wi-Fi 2.4 GHZ and a 32 bit processing unit.

**Figure 8:**
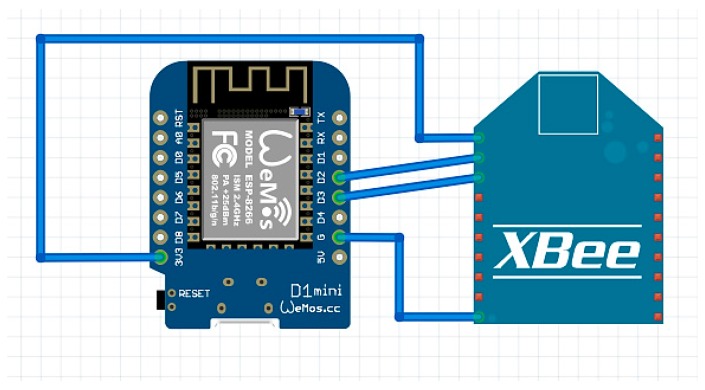
iAQ Gateway.

The iAQ Gateway uses only wireless technologies for communication between nodes as well as for Internet connection. It receives data from iAQ Sensor via XBee technology and then uses Wemos integrated Wi-Fi to send data to a MySQL database using Restfull webservices. The schematic and connections used in iAQ Gateway are described in Figure 8.

### 2.5. Software

The firmware of the iAQ Sensor and iAQ Gateway was implemented using the Arduino platform language in the IDE ARDUINO. It belongs to the C-family programming languages.

The iAQ Web was developed in PHP and MySQL database. Web services that allow data collection are also built in PHP [37].

iAQ Mobile was built for an Android system using the IDE AndroidStudio and has the minimum requirements API 15: Android 4.0.3 Ice Cream Sandwich.

## 3. Results and Discussion

**Figure 9:**
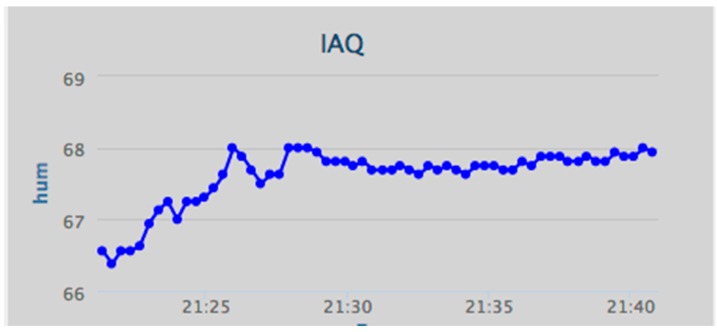
Data visualization: relative humidity (%).

**Figure 10:**
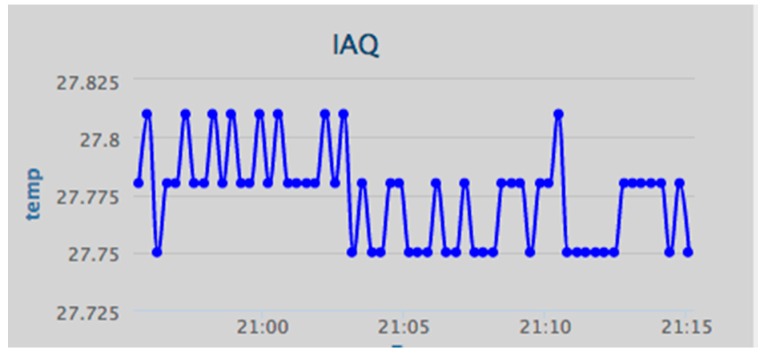
Data visualization: temperature (°C).

**Figure 11:**
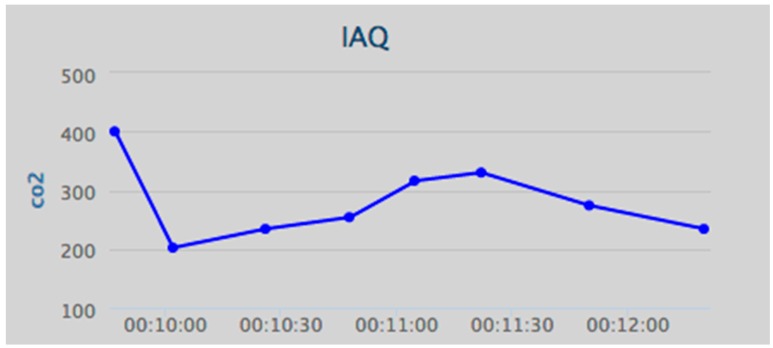
Data visualization: carbon dioxide (CO_2_) concentration (ppm).

Humans will often be the integral parts of the IoT system; therefore, IoT will affect every aspect of human lives [38], and IoT technologies provide many benefits to the healthcare domain in activities such as the tracking of objects, patients and staff, the identification and authentication of people, automatic data collection, and sensing [39]. Considering the importance of the air quality in indoor environments, the iAQ web portal allows the user to view the data as numerical values or as a graph. Some of the charts in hand in the web application are shown below, such as the relative humidity chart (Figure 9), the air temperature chart (Figure 10), and the CO_2_ chart (Figure 11).

The iAQ Mobile application has several significant advantages because in fact 56% of American adults are now smartphone owners [40]; for example, in The Netherlands, 70% of the general population and over 90% of adolescents own a smartphone [41]. Another important reason for the development of mobile applications is that a study proposed in [42] concludes that unlocked usage of phones constitute on average 86 min per day (median 58 min). These arguments define the importance of mobile devices. Another study proposed in [43] observed that different usage patterns may apply to different locations for each user, and smartphones were used considerably, even when users were close to PCs that were accessible to them, an argument that contributes to the motivation of creating the mobile application.

**Figure 12:**
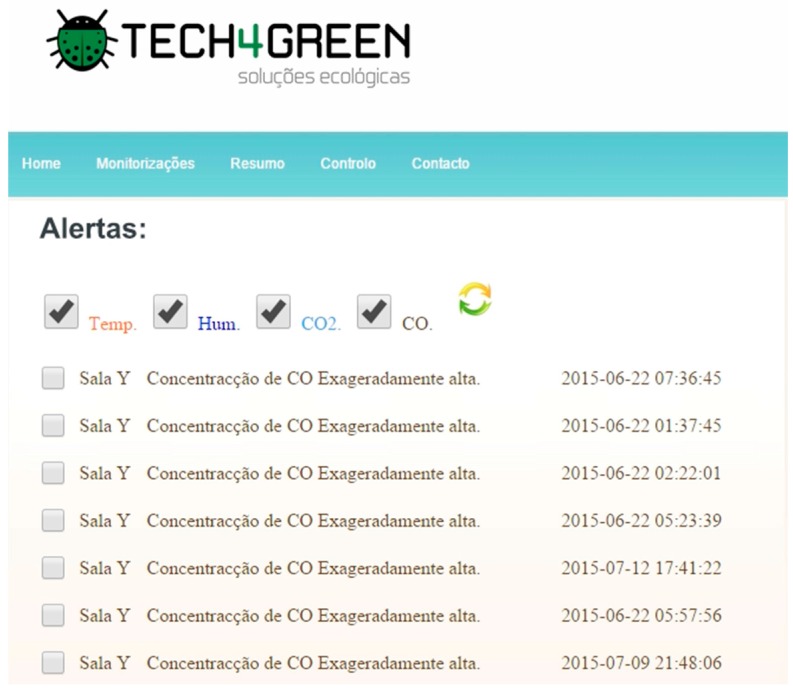
iAQ notifications system.

Another important feature of the application is the notification system that can be accessed both on the web portal and the mobile application. Figure 12 shows the Web portal notifications which consists of a list of poor air quality notifications. This data is generated when a monitored parameter exceeds the specified minimum or maximum limit set to reference air quality.

IoT gives a perfect platform to ubiquitous healthcare, using, for example, wearable sensors for uploading data to servers and smartphones for communication, along with Bluetooth for interfacing sensors measuring physiological parameters [44]. IoT systems and AAL will continue side by side, mutually contributing scientific advances in assisted living, thereby allowing for a reduction in the cost of assisted living systems. The iAQ system presented by the authors aims to be an air quality monitoring solution for healthcare that provides indoor air quality data for the user, caregiver, and doctor in real time, assuming itself as a solution to ambient assisted living based on the paradigm of the IoT. The authors aim to develop solutions based on the iAQ system for specific environments such as residential institutions, industrial environments, offices, and hospitals.

This system has several advantages over existing systems for indoor air quality monitoring, such as the low cost of implementation and installation due to its modularity and its size. The iAQ system uses wireless technologies not only for communication between the iAQ Sensors and iAQ Gateway but also for communication between the iAQ Gateway and the Internet connection. This factor decreases the cost of implementation and installation compared with other systems that incorporate Ethernet connection between the gateway and the Internet. This system can also be easily configured to use only as many iAQ Sensors as necessary due to their modularity. Compared with other solutions [45,46], this system is distinguished through ease of access to real-time air quality data using the website and the mobile app to receive real-time notifications.

The iAQ system provides a maximum transmission range of 27 m between the end-device and the coordinator. Compared with other systems, specifically the indoor air quality monitoring system based on WSNs, technology proposed in [11,47], which provides 10 m and 20 m of maximum transmission range between nodes, respectively, the iAQ system provide better transmission range results. 

The lifetime and energy consumption of the iAQ system’s WSN is an important issue and challenge to manage and solve as a future project. In order to improve the power consumption of the iAQ Sensor, the air quality MOS (Metal Oxide Semiconductor) and NDIR (Near-infrared) types should be replaced by electrochemical sensors, as they are more energy-efficient as proposed in [48]. The selection of the sensors was made focusing on the cost of the system, since the main objective was to test the functional architecture of the system. In the future, to improve the battery lifetime electromechanical sensors will be implemented in the system.

The goal of the authors is that in the future we can face indoor air quality monitoring as a key factor in order to improve the quality of life and health of the population.

## 4. Conclusions

This paper presents iAQ, an air quality monitoring system based on IoT architecture for ambient assisted environments. iAQ was developed using open-source technologies and low-cost sensors. This system incorporates five sensors, but other sensors can be added for specific parameter monitoring.

The results show that indoor air quality might be, to a great degree, distinctively contrasted with what is normal for a quality living environment but represents a significant contribution to indoor environmental studies, as it presents itself as a solution for easy installation, is modular, and allows easy access via web portal or from a smartphone to the indoor air quality data while also notifying the user of critical situations of poor air quality.

IoT systems and AAL will continue side by side, mutually contributing scientific advances in assisted living, thereby allowing for a cost reduction in assisted living systems; however, despite the many technologic advances, some difficulties in the construction of assisted living systems continue to exist, in particular, the privacy, confidentiality, and security of such systems. IoT continues to have several QoS (Quality of Service) issues such as availability, reliability, mobility, performance, scalability, and interoperability. Security and data privacy are the main challenges of IoT. In the future, iAQ should address these problems of the IoT paradigm, creating tools in order to respond to these problems.

Another great advantage of this system is the use of wireless technology for communication between the iAQ Sensor nodes and iAQ Gateway, and wireless connection to the Internet by using Wi-Fi incorporated in ESP8266 as well as a mobile app for Android users to view relevant information and receive notifications anytime and anywhere. The mobile application has the advantage of the use of remote real-time notifications that may help the user to maintain good indoor air quality in a home to increase the occupant’s health, productivity, and well-being.

Future work is planned to conduct more tests with more than three modules in order to improve the performance of the communication architecture, in terms of latency, throughput, packet loss, etc., and to discover difficulties that arise when a great number of nodes are used at the same time to communicate [49]. More tests are planned to conduct to the WSN architecture with special attention paid to power consumption in order to improve battery lifetime and to decrease the influence of gas sensors in power consumption [50]. This real-time indoor air quality monitoring system can also be used to support the building manager for the proper operation and maintenance in providing not only a safe and healthy workplace, but also a comfortable and productive one.

## Figures and Tables

**Table 1:** WSN consumption (mA).

Node State	iAQ Sensor	iAQ Gateway
Sleeping	108	54
Awake (Transmitting)	274	247
Awake (Receiving)	129	139
